# From dark modes to topology: light-induced skyrmion generation in a plasmonic nanostructure through the inverse faraday effect

**DOI:** 10.1515/nanoph-2025-0096

**Published:** 2025-06-18

**Authors:** Xingyu Yang, Ye Mou, Bruno Gallas, Sébastien Bidault, Mathieu Mivelle

**Affiliations:** Sorbonne Université, CNRS, Institut des NanoSciences de Paris, INSP, F-75005 Paris, France; School of Electronic and Information Engineering, Ningbo University of Technology, No. 201, Fenghua Road, Jiangbei District, Ningbo, Zhejiang, China; Institut Langevin, ESPCI Paris, Université PSL, CNRS, F-75005 Paris, France

**Keywords:** skyrmion, plasmonic nanoantenna, inverse Faraday effect, nanophotonics, light matter interactions

## Abstract

Skyrmions are topological structures characterized by a winding vectorial configuration that provides a quantized topological charge. In magnetic materials, skyrmions are localized spin textures that exhibit unique stability and mobility properties, making them highly relevant to the burgeoning field of spintronics. In optics, these structures open new frontiers in manipulating and controlling light at the nanoscale. The convergence of optics and magnetics holds therefore immense potential for manipulating magnetic processes at ultrafast timescales. Here, we explore the possibility of generating skyrmionic topological structures within the magnetic field induced by the inverse Faraday effect in a plasmonic nanostructure. Our investigation reveals that a gold nanoring, featuring a dark mode, can generate counter-propagating photocurrents between its inner and outer segments, thereby enabling the magnetization of gold and supporting a skyrmionic vectorial distribution. We elucidate that these photocurrents arise from the localized control of light polarization, facilitating their counter-propagative motion. The generation of skyrmions through the inverse Faraday effect at the nanoscale presents a pathway towards directly integrating this topology into magnetic layers. This advancement holds promise for ultrafast timescales, offering direct applications in ultrafast data writing and processing.

## 1 Introduction

In recent years, the field of condensed matter physics has witnessed a surge of interest in a class of intriguing topological structures known as skyrmions [1]. These nanoscale whirlpools of magnetic order, originally conceived as solutions to equations of nuclear physics by Tony Skyrme in the early 1960s [2], have emerged as promising candidates for revolutionizing information storage and processing technologies [3]. In magnetic materials, skyrmions are localized spin textures that exhibit unique stability and mobility properties, making them highly relevant to the burgeoning field of spintronics [4]. The distinctive feature of skyrmions lies in their nontrivial topology, characterized by a winding configuration of spins that provides a quantized topological charge to these entities [5]. Unlike traditional magnetic domains, skyrmions possess a particle-like nature and can exist as individual entities or form periodic arrays in magnetic materials [6]. Their stability at nanoscale dimensions and the ability to manipulate them with low-energy currents have positioned skyrmions as potential building blocks for next-generation magnetic memories and logic devices [7].

The allure of skyrmions extends beyond the realm of condensed matter physics, permeating diverse fields of research with their unique topological characteristics and dynamic behaviors. They have, for instance, manifested in unexpected domains such as optics [8], [9], [10], [11], [12], opening new frontiers in the manipulation and control of light at the nanoscale. In fact, the unique topological features of skyrmions, characterized by their swirling vectorial configurations, introduce intriguing possibilities for tailoring optical properties and creating innovative devices in photonics. Moreover, in recent years, researchers have explored the interaction between skyrmions and light, uncovering fascinating phenomena that bridge the fields of magnetism and optics [13]. The integration of skyrmions into photonic structures also holds promise for developing all-optical information processing devices [14], and quantum optics [15]. Skyrmions, with their nanoscale dimensions and stability, can serve as dynamic elements in optical circuits, enabling the transmission and manipulation of information through the controlled motion of these magnetic textures. This burgeoning field of research not only extends the functionalities of existing photonic technologies but also introduces novel paradigms for the design of next-generation optical devices.

Here, we propose to go one step further in the interaction between light and magnetism at the nanoscale by developing a model of plasmonic nanostructures enabling the generation of a magnetic field by inverse Faraday effect (IFE) bearing the topological structure of a skyrmion. The IFE is a magneto-optical process enabling the magnetization of matter by optical excitation. While known since the 1960s [16], [17], [18], [19], [20], this physical phenomenon has recently garnered attention due to advancements in nanophotonics and ultrafast optics [21], [22], [23], [24], [25], [26], [27], [28], [29], [30], [31], [32], [33]. The manipulation of light at the nanoscale, specifically with regard to its polarization, gradients, and amplitude, has been demonstrated to yield ultrafast and intense magnetic fields at this scale [24], [26], [27]. These properties offer avenues for manipulating magnetic processes at ultrafast timescales and nanoscopic spatial dimensions [34], [35], [36]. In this paper, we establish that a ring-shaped plasmonic nanostructure generates a skyrmionic distribution of magnetic fields through IFE. This novel behavior arises from the production of two drift countercurrents within the metal of the ring, notably in its inner and outer segments. These opposite drift currents result from the manipulation of the polarization of light around the plasmonic antenna, achieved through the specific excitation of the anti-bonding mode of the plasmonic nanoantenna.

The significance of these findings lies in their potential to facilitate the implementation of skyrmionic topological structures within a magnetic layer through direct magnetic action and potentially at ultrafast timescales. This breakthrough, therefore, opens avenues for developing next-generation magnetic memory and logic devices.

## 2 Results

**Figure 1:**
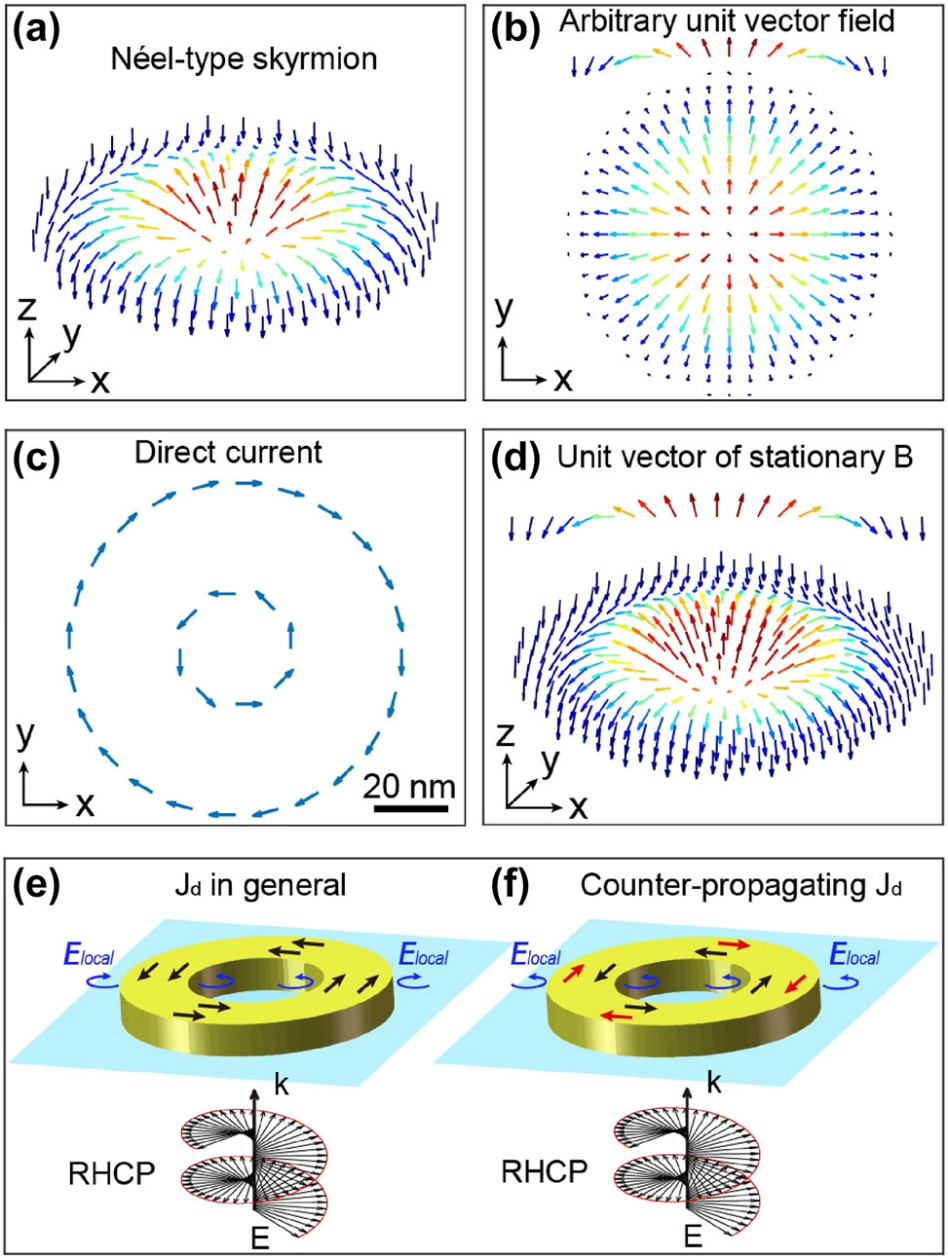
Description of a Néel-type skyrmion. (a) 3D and (b) normal perspective of unit vector distributions of a Néel-type skyrmion. The arrows represent the orientation of the vectors, and the colors are the amplitude of their Z component. (c) Distribution of direct electric currents in an XY plane, required for the realization of (d) a Neel-type magnetic skyrmion distribution for a rotation of the vector in the XZ and YZ planes. (e) and (f) Schematic description of the plasmonic nanostructure considered in this study to generate a skyrmionic distribution by IFE: a gold nanoring is placed on a glass substrate and excited by a right circularly polarized plane wave incident from the substrate side. Two cases are considered: drift currents (***J**_**d**_*) generated by IFE propagate (e) in the same direction and (f) in opposite directions between the inner and outer parts of the nanoring. The blue arrows represent the local polarization required to generate the drift currents shown in the drawing, black and red arrows indicate the direction of drift currents.

A skyrmion can exhibit diverse topological structures [5]. However, our focus in this article centers exclusively on the Néel type. Illustrated in Figure 1a and b, we outline the vector distribution of the magnetic field within this specific manifestation of a skyrmionic structure. This type is characterized by a continuous rotation of the magnetic field in the plane of the skyrmion, extending seamlessly from its center to its periphery, forming an intricate swirling pattern [37]. Figure 1c and d depict one method of reaching such a vectorial distribution in the magnetic field, involving the generation of two counter-propagating currents with different radii.

For the creation of this intricate vectorial distribution within a nanoscale magnetic field, we propose the use of a ring-shaped plasmonic nanoantenna, as illustrated in Figure 1e and f, generating counter-propagating drift currents through IFE, as illustrated in Figure 1f. To this end, the nano-ring is positioned on a glass substrate and excited by a right-circularly polarized plane wave incident from the substrate side (Figure 1e and f).

The theoretical description of the drift currents (***J**_**d**_*) induced by IFE in a metal has been extensively documented [38], [39]. The ensuing Equation describes this phenomenon:

(1) $$J_{d}=\frac{1}{2en}Re\left(\left(-\frac{\nabla \cdot \left(\sigma_{\omega }E\right)}{i\omega }\right)\cdot {\left(\sigma_{\omega }E\right)}^{*}\right)$$

Here *e* represents the charge of the electron (*e* < 0), *n* is the charge density at rest, *σ*_*ω*_ denotes the dynamic conductivity of the metal, and ***E*** corresponds to the optical electric field. It is important to note that Equation (1) gives the time averaged drift current, which therefore behaves as a direct current (DC) within the metal. This expression for drift currents incorporates several contributions to IFE, including a microscopic contribution [38] and a macroscopic contribution [39]. Both of these contributions are detailed in the Supporting information, and using the full equation of ***J**_**d**_* allows for a comprehensive consideration of both. However, a detailed study is included at the end of this work to distinguish these contributions in the generation of the skyrmionic topology in the magnetic field through the IFE.

Using these currents and employing the Biot and Savart equation (Equation (2)), the vectorial distribution of the magnetic field can be computed in the space surrounding the nanoantenna. Also, because the current is DC, the associated magnetic field is constant in time.

(2) $$B=\frac{\mu_{0}}{4\pi }∭\frac{J_{d}\times r}{{\left|r\right|}^{3}}dV$$

Equation (1) implies that the prerequisite for inducing counter-propagating currents on opposing sides of the nanoring is the presence of light, both inside and outside, carrying the same type of polarization helicity, as depicted in Figure 1f.

**Figure 2:**
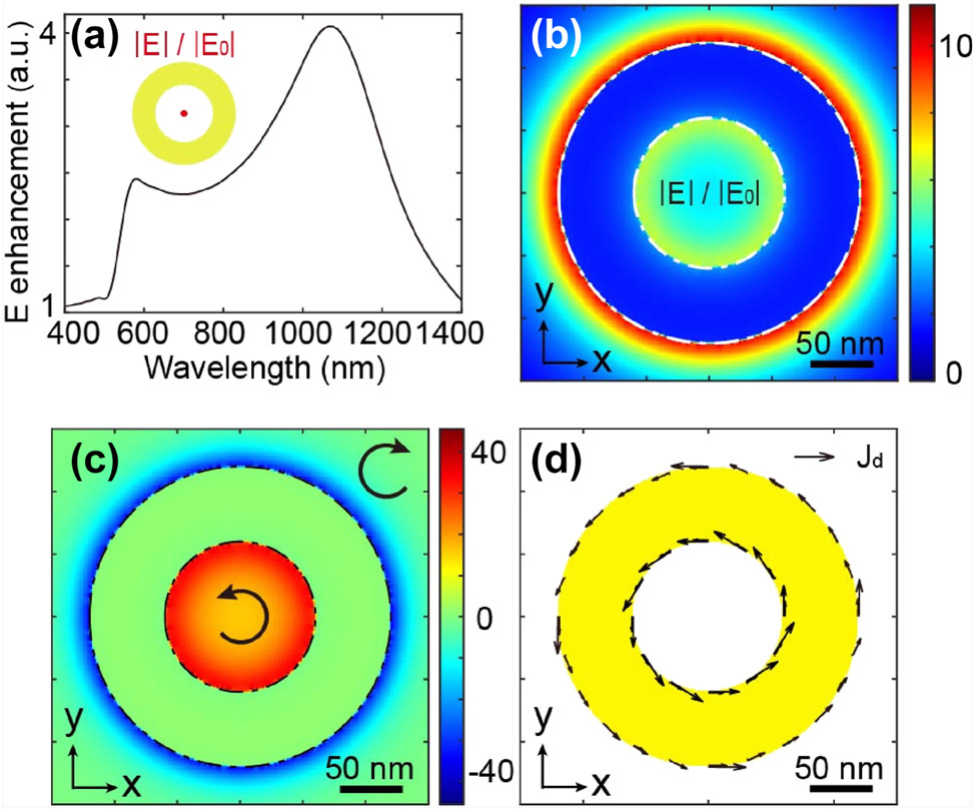
Description of the physical behavior of the nanoring. (a) Spectral response in terms of electric field enhancement at the XYZ center of a nanoring with an inner diameter of 120 nm, outer diameter of 240 nm, and thickness of 30 nm. Spatial distribution in an XY plane at the Z center of the nanoantenna for (b) electric field enhancement normalized by the incident wave, (c) spin density, and (d) drift currents. It is important to note that for all quantitative measurements in this study, the electric field amplitude used in our simulations was set to 1 V/m.

Figure 2a illustrates the spectral response of the electric field enhancement at the XYZ center of a nanoring, featuring an inner diameter “d” of 120 nm and an outer diameter “D” of 240 nm with thickness 30 nm. Notably, for these dimensions, the structure exhibits two resonances in its spectral response, with one being weaker around 570 nm and the other more pronounced at approximately 1,100 nm. Let’s first delve into the characteristics of the stronger resonance. In Figure 2b, the electric field distribution in an XY plane at the Z center of the structure is outlined at a wavelength *λ* of 1,100 nm. The field distribution indicates a notable increase in the electric field both inside and outside the nanoring at this specific wavelength.

Moving forward, Figure 2c shows the spin density distribution in the same plane as the field in Figure 2b. The spin density, as defined by Equation (3), represents a vectorial physical quantity that illustrates the polarization state of light within a given plane. Figure 2c displays the Z component of the spin density describing the helicity of light in the XY plane. This density can reach positive or negative values, corresponding to right or left elliptical polarizations. Specifically, a positive spin density in our reference system implies a right helicity, a negative spin density corresponds to a left helicity, and a zero density denotes linear polarization. In the far field, the spin density is constrained between −1 and 1, with −1 indicating left circular polarization and 1 denoting right circular polarization. However, in the near field, when normalized by the incident intensity ∣*E*_0_∣^2^, the spin density can reach significantly larger values due to the enhanced fields, giving rise to the concept of super-circular light [31], drawing an analogy with super-chiral light [40].

(3) $$s=\frac{1}{{\left|E_{0}\right|}^{2}}Im\left(E^{*}\times E\right)$$

From Figure 2c, it is clear that the spin densities within and outside the ring exhibit opposite signs. This local light polarization state does not provide the prerequisite for counter-propagating drift currents, as explained earlier and detailed in Figure 1e and f. Consequently, in this scenario, the currents will flow in the same direction, as illustrated in Figure 2d, and they will not generate a skyrmionic structure through IFE.

**Figure 3:**
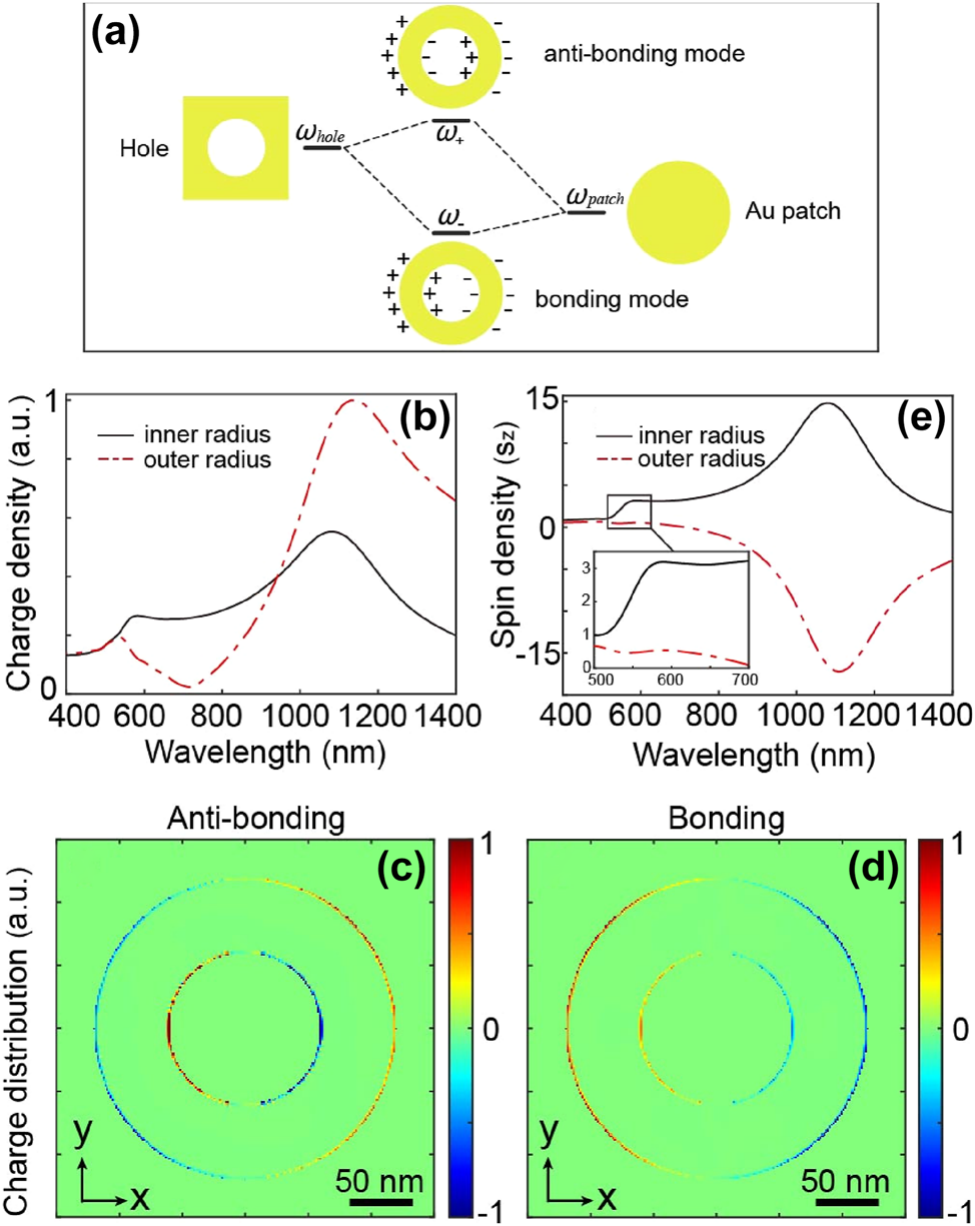
Multipolar behavior of the nanoring. (a) A nanoring can be seen as the combination of two distinct structures, namely a nanohole and a nanopatch. Each of these structures has its resonance mode, and their coupling results in a structure itself carrying two resonance modes, one bright, known as bonding, at a lower frequency, and the other dark, known as anti-bonding, at a higher frequency. (b) Spectral response in terms of electron density for the inner (black line) and outer (red dashed lines) parts of the metal. Spatial distributions of electron densities in an XY plane at the Z center of the nanoring for (c) the anti-bonding mode and (d) the bonding mode. (e) Spectral response in terms of spin density for the inner (black line) and outer (red dashed line) parts of the nanoring. A zoom of the portion corresponding to the anti-bonding mode is shown in the inset.

Nevertheless, these annular plasmonic structures are known in the literature for supporting a diversity of modes [41], [42], [43]. Specifically, a nanoring can be seen as a combination of two distinct structures: a circular nano-aperture and a nanodisk, each exhibiting a resonance mode (Figure 3a and Figure S1). The combination of these structures results in a nanoring with the ability to sustain two distinctive coupled modes (Figure 3a): a bonding mode (commonly referred to as bright) and an anti-bonding mode (commonly referred to as dark). The manifestation of these modes becomes clear when studying the spectral response of the nanoantenna with respect to the charge density (Figure 3b) within the inner and outer metallic components of the antenna. Charge density distributions corresponding to the bonding and anti-bonding modes identified in Figure 3b are presented in Figure 3c and d, respectively. As anticipated, the charge densities between the inner and outer regions of the antenna exhibit an out-of-phase relationship for the anti-bonding mode, while they are in phase for the bonding mode.

Upon inspecting the spectral response regarding spin densities for the inner and outer segments of the nanoring (Figure 3e), it is evident that polarizations are opposite in the case of the bonding mode (at *λ* = 1,100 nm in Figure 3e). In contrast, for the anti-bonding mode, the spin densities exhibit the same sign within a specific wavelength range (at *λ* = 570 nm in Figure 3e).

**Figure 4:**
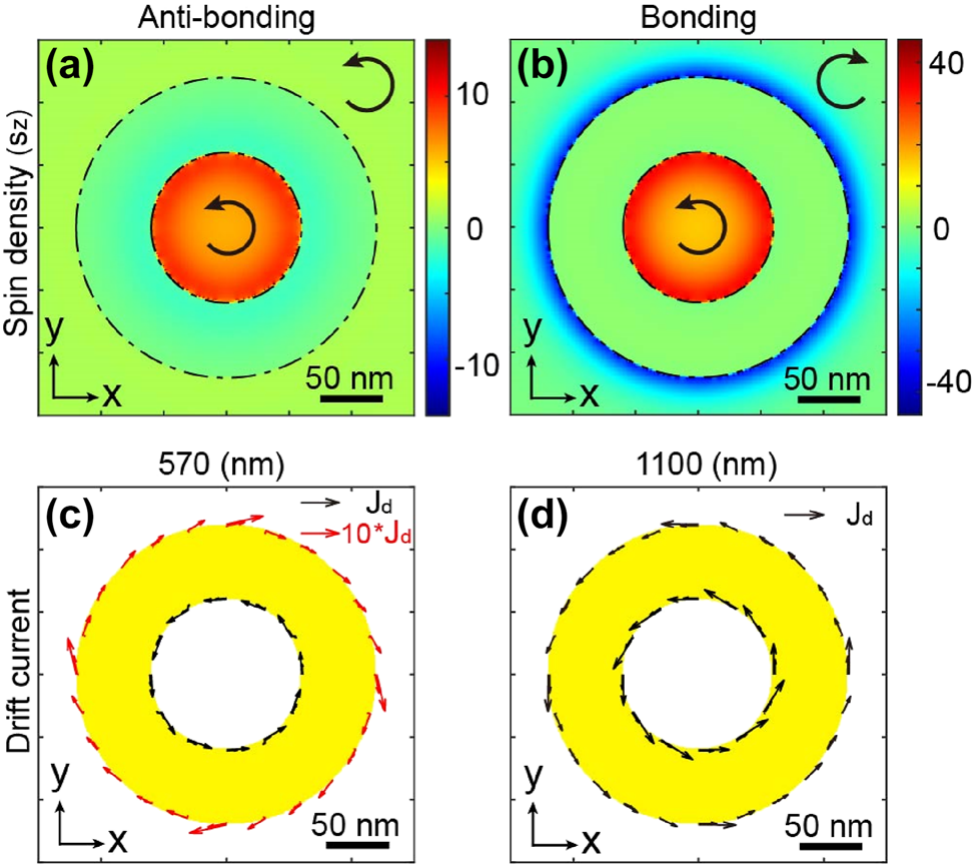
Comparison of dark and bright modes in the nanoring. Spatial distributions of spin densities in an XY plane at the Z center of the plasmonic nanostructure for (a) the anti-bonding mode (*λ* = 570 nm) and (b) the bonding mode (*λ* = 1,100 nm). Arrows represent the helicity of light. Spatial distributions of drift currents in an XY plane at the Z center of the nanoring for (c) the anti-bonding mode and (d) the bonding mode. The length of the arrows represents the relative amplitude of the generated photocurrents.

To illustrate this difference, Figure 4a and b present spin density distributions in an XY plane at the Z center of the nanoring for the two modes inherent to this antenna. The contrasting polarizations are clearly visible in these depictions. Subsequently, Figure 4c and d exhibit the drift currents corresponding to the spin densities in Figure 4a and b, calculated using Equation (1). In the bonding mode, we observe that the drift currents propagate in the same directions within the inner and outer regions of the nano-antenna. In contrast, in the case of the anti-bonding mode, the currents exhibit counter-propagation on each side of the nanoring.

Consequently, this dark mode is anticipated to facilitate the generation of a skyrmionic topological distribution through IFE, as illustrated in Figure 1c and d. The dissimilarity in local polarization between these two modes is found in the dipolar nature of the bonding mode and the coupling between two dipolar modes within the anti-bonding mode. Comprehensive information on these divergent effects is provided in the Supporting information.

**Figure 5:**
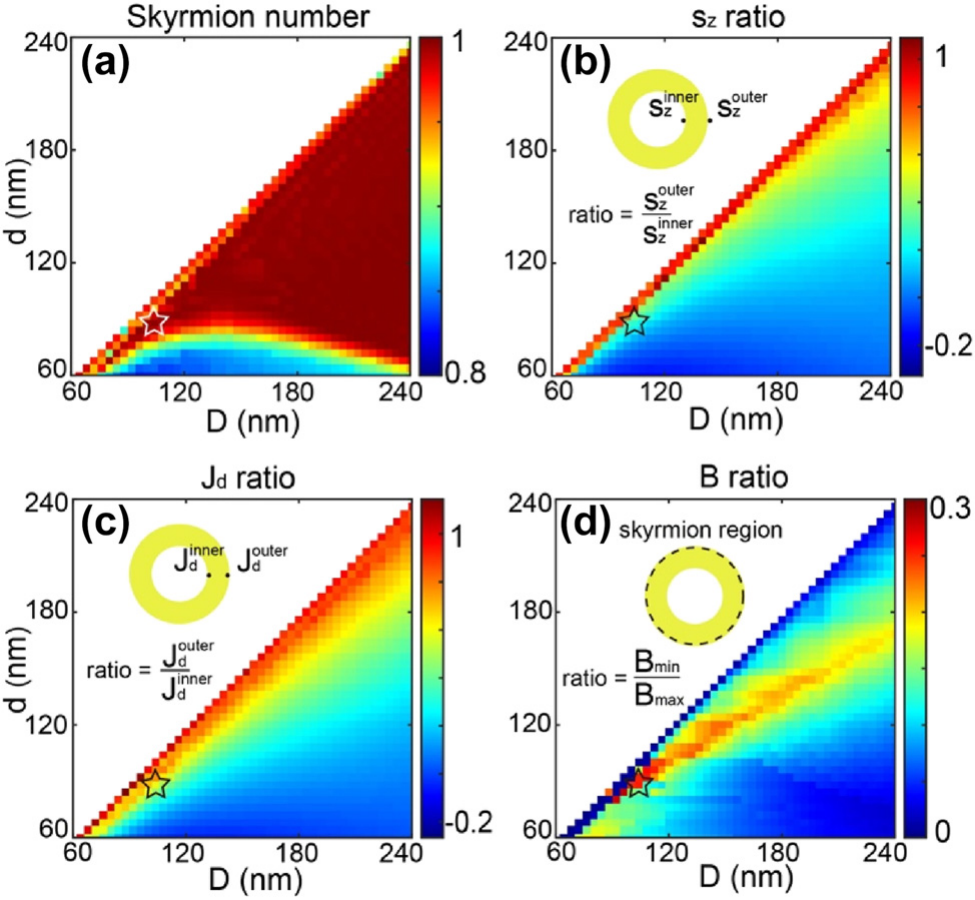
Study of skyrmion generation by IFE for different inner “d” and outer “D” nanoring diameters, with a 30 nm gold thickness and excitation at *λ* = 600 nm. (a) Skyrmion number values. (b) Ratio of spin density values along Z between the inner and outer parts of the plasmonic nanostructure. (c) Ratio of drift currents (absolute values) associated with the spin densities shown in (b) between the inner and outer parts of the nanoring. (d) ratio between the maximum and minimum magnetic field amplitudes generated from the drift currents shown in (c) within the skyrmionic region of the plasmonic nanoantenna. The star highlights the dimensions considered in Figure 6.

Having identified the physical process responsible for the generation of counter-propagating drift currents, an opportunity arises to investigate the parameters governing this phenomenon. Our focus centers on determining a configuration with dimensions capable of inducing drift currents of comparable magnitudes in both the internal and external regions of the nanoring, resulting in a uniform magnetic field amplitude. Figure 5 explores the set of parameters “d” and “D” to achieve a skyrmionic vector distribution of the magnetic field. In Figure 5a, the skyrmion number of the nanoring is presented for excitation at a wavelength of 600 nm, considering various values of “d” and “D” with a gold thickness of 30 nm. The skyrmion number “*Q*”, an integral topological invariant used to characterize the winding of spins in the magnetic texture, is defined as an integer and is associated with the topology of the spin configuration within the skyrmion. “*Q*” is defined by unit vector ***u***, expressed as:

(4) $$Q=\frac{1}{4\pi }\iint u\cdot \left(\frac{\partial u}{\partial x}\times \frac{\partial u}{\partial y}\right)dxdy$$

**Figure 6:**
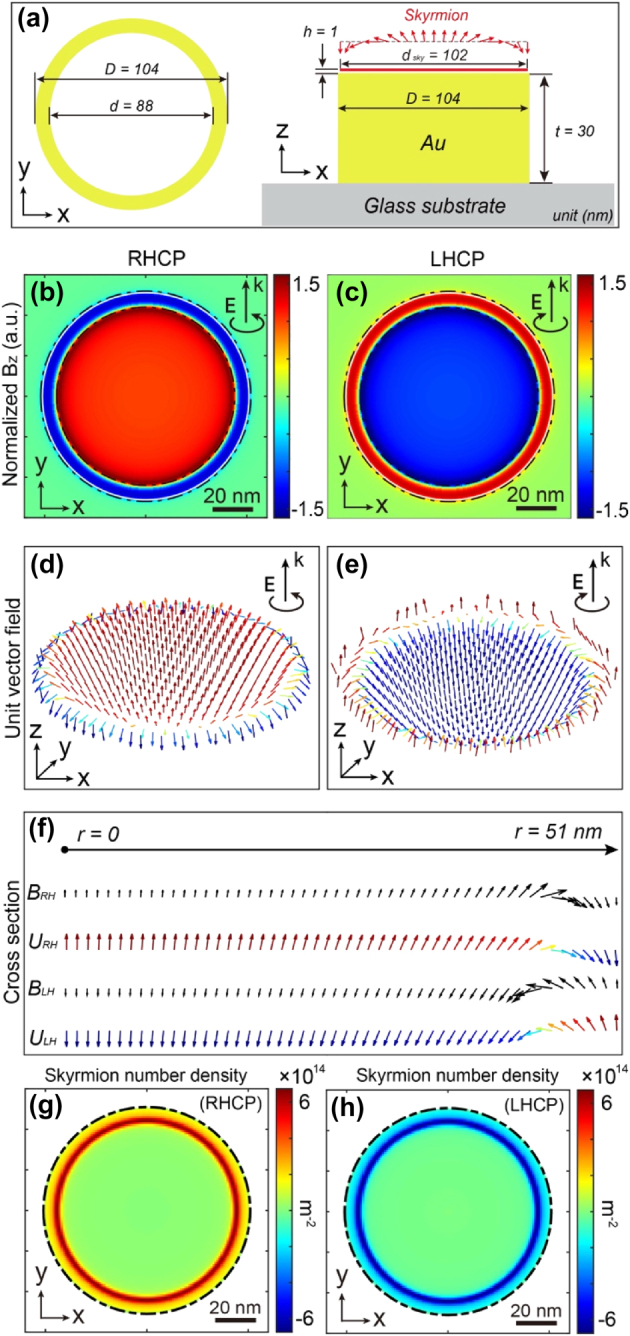
Example of a nanostructure generating a perfect Néel-type skyrmion for excitation at *λ* = 600 nm. (a) Schematic representation of a nanoring enabling the generation of a Néel-type skyrmion by IFE. Its dimensions include an inner diameter of 88 nm, an outer diameter of 104 nm, and a 30 nm gold thickness. Spatial distribution of the normalized magnetic field along Z generated by IFE in an XY plane 1 nm above the nanoring for (b) right and (c) left circularly polarized optical excitation, normalized by the **B** field amplitude at the center. (d) and (e) 3D vectorial distribution of unit vector **B** represented in (b) and (c), respectively. The black circular arrow at the right top of the figures indicates the polarization direction of the incident wave. (f) Line cut of vectorial distributions of magnetic fields in amplitudes (B) and normalized (U) from the center to the edge of the nanostructure for right-handed (RH) and left-handed (LH) circular polarizations. (g) and (h) Show the skyrmion number density distribution for right and lefthanded circular polarizations, respectively, in the same plane as (b) and (c).

In our study, the integration region for the skyrmion number extends from the center of the ring to the outer radius where the out of plane component of the magnetic field reaches its maximum on the outer side of the ring. A vector field distribution closest to a perfect Neel-type skyrmionic topology is observed when the skyrmion number, *Q*, approaches ±1. The sign of *Q* dictates the up or down orientation of the magnetic field at the center of the antenna. Figure 5a illustrates that numerous pairs of parameters, “d” and “D”, can yield a value of *Q* close to 1, indicating that various physical parameters can be manipulated to achieve a skyrmion (See the Supplementary information for an additional example of a structure capable of generating the same topology). Skyrmion numbers that deviate from unity (e.g., *Q* ≈ 0.8) arise when the inner and outer currents flow in the same rather than opposite directions, giving a negative inner/outer current ratio (Figure 5c). In these geometries the dark mode is not excited, and a skyrmionic topology cannot form. Despite the magnetic field’s vector distribution taking on a skyrmionic topology, our objective was to develop a plasmonic nanoantenna with a relatively uniform magnetic field amplitude in this vectorial distribution. To this end, Figure 5b presents the ratio of spin density amplitudes between the inner and outer segments of the nanoring for different diameter values. This ratio aligns well with the ratio of drift countercurrents between the inner and outer metal components, as depicted in Figure 5c. Using these drift currents, we calculate the ratio between the maximum and minimum magnetic fields on either side of the nanoring walls, as illustrated in Figure 5d. This figure provides a range of parameters that enable the skyrmionic distribution to exhibit a relatively homogeneous magnetic field amplitude.

Building upon these findings, Figure 6 depicts the vectorial distribution of the magnetic field generated by IFE for a nanostructure derived from our simulations, considering two types of optical excitations: right-circularly polarized and left-circularly polarized. The selected nanoantenna is showcased in Figure 6a, having a thickness of 30 nm, an inner diameter of 88 nm, and an outer diameter of 104 nm. The ring dimensions marked with a star in Figure 5a were chosen arbitrarily from the pool of geometries that do support a skyrmion; this particular set yields the most uniform magnetic field amplitude over the area of interest. Figure 6b and c illustrate the distribution of the Z-component of the optically induced magnetic field, normalized by the magnetic field amplitude in the center, in case of right- and left-circular polarizations, respectively. Consistent with the IFE principle, these vector distributions are perfectly inverted in orientation, as shown in Figure 6d and e, presenting these fields in 3D at 1 nm from the gold surface of the antenna.

Figure 6d and e present the unit vector distributions of **B**. As observed, a skyrmionic topological distribution manifests on the surface of this antenna. The magnetic field is perpendicular to the surface at the center of the antenna, progressively rotating within the skyrmion plane from the center to the periphery of the antenna, forming a swirling pattern that completely reverses at the edge of the nanoantenna. To illustrate this vectorial motion in space, Figure 6f displays the orientation of the magnetic field and its unit vector for a linecut from the center to the edge of the antenna. The spatial inversion of the magnetic field is evident, with the magnetic field maintaining the same sign over the entire nanoantenna, and a flip of the magnetic field occurring at the end of the nanostructure. To make this clearer, Figure 6g and h display the skyrmion number density distribution (*S*(*x,y*)) defined in Equation (5),

(5) $$S\left(x,y\right)=\frac{1}{4\pi }u\cdot \left(\frac{\partial u}{\partial x}\times \frac{\partial u}{\partial y}\right)$$

which quantifies the local concentration of topological charge. Regions where *S*≠0 indicate the characteristic twisting of a skyrmion. In our system the nonzero values are localized near the nanoring, confirming that the topology is generated in that vicinity.

This outcome is particularly noteworthy, aligning with the observed behavior in many skyrmionic spin structures found in magnetic materials [44]. These characteristics could facilitate the implementation of such vectorial structures in magnetic materials using plasmonic IFE. Additionally, the composition of magnetic materials defines the physical size of skyrmionic topologies. Thus, the ability to adjust the size of the vectorial distributions with this symmetry by changing the dimensions of the plasmonic nanostructures would be a valuable advantage for the all-optical generation of skyrmions. A practical route toward exploiting and characterising these skyrmionic fields would be to map the vector distribution of the magnetic field – or the induced magnetic moment – inside an adjacent magnetic layer that can host such textures. A nearfield magnetometry technique would then be required. An atomic force microscope equipped with a single nitrogen-vacancy (NV) center in a diamond tip would be then ideally suited: it combines high magnetic field sensitivity, nanometers spatial resolution, and full vector mapping capability.

Now that we have demonstrated how a plasmonic nanoring structure can generate counter-propagating currents, enabling the formation of a skyrmionic topology in the magnetic field, let us turn our attention to the various microscopic and macroscopic contributions of the IFE to this topology. As described in the Supplementary materials, the equation for the drift currents ***J**_**d**_* can also be expressed as:

(6) $$J_{d}=\nabla \times M+\Gamma =J_{M}\;+\;J_{r}$$

with

(7) $$\nabla \times M=-\frac{j}{4e\left\langle n\right\rangle \omega }\nabla \times \left(\sigma^{*}E^{*}\times \sigma E\right)$$

and

(8) $$\Gamma =\frac{1}{4e\left\langle n\right\rangle \omega }\left[j\left(\sigma^{*}E^{*}\cdot \nabla \right)\sigma E+c\cdot c\cdot \right]$$

where ***M*** represents the magnetization, ∇ × ***M*** denotes the magnetization currents ***J**_**M**_*, and **Γ** represents the ponderomotive currents ***J**_**Γ**_*. As described in the Supplementary information, and as identified in the case of a uniform metal layer excited by a Gaussian beam, explained by R. Hertel [39], the magnetization ***M*** and the magnetization currents ***J**_**M**_* are two sides of the same phenomenon and produce the same magnetic moment. However, the ponderomotive currents exhibit a distinct behavior, which can potentially oppose that of the magnetization currents (see Supplementary information) [39].

**Figure 7:**
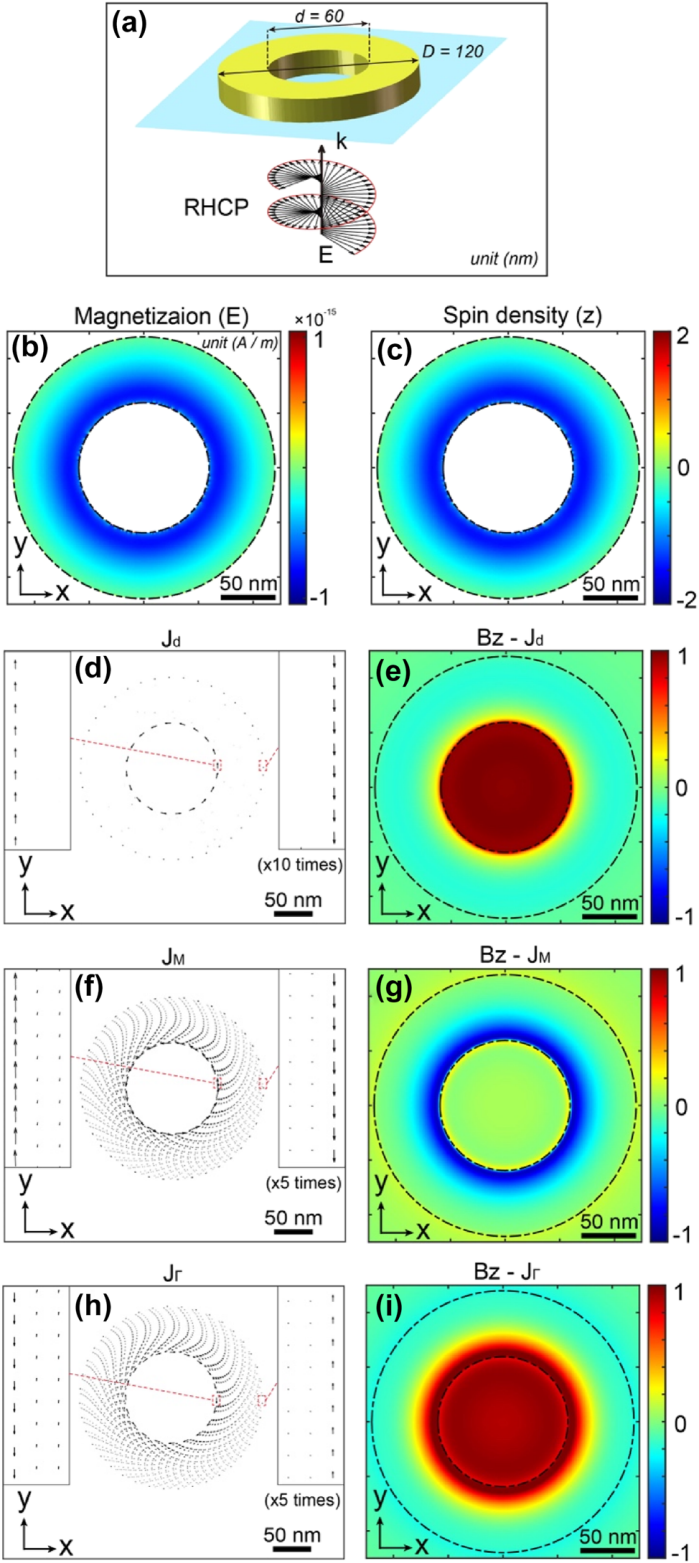
A gold nanoring with an inner diameter of 60 nm, outer diameter of 120 nm and a thickness of 30 nm, placed on a glass substrate and excited by a right-handed circularly polarized plane wave at a wavelength of 570 nm, under normal incidence. (a) Schematic representation of the system. Distributions in the central plane (Z) of the nanoring for: (b) magnetization ***M***, (c) spin density, (d) drift currents ***J**_**d**_*, (f) magnetization currents ***J**_**M**_*, and (h) ponderomotive currents ***J**_**Γ**_*. (e), (g), and (i) show the corresponding magnetic field distributions at the nanoring’s surface for the currents displayed in (d), (f), and (h), respectively.

Figure 7 illustrates these various contributions to the magnetization and the distribution of the magnetic field generated by the IFE. Figure 7a shows a schematic of the nanoring from Figure 4a, excited by a right-handed circularly polarized plane wave, which we use to differentiate the various contributions of the IFE. Figure 7b represents the distribution of the magnetization ***M***, linked to the microscopic part of the IFE, in the XY plane at the center (Z) of the nanoring. As shown, this magnetization is negative throughout the nanoring, with a total contribution corresponding to a magnetic moment of −4.7767e^−37^ A m^2^ (equal to the spatial sum of the moments shown in Figure 7b). This uniformity in the magnetization sign is a result of the single helicity of the light within the metal of the nanoring, as illustrated in Figure 7c via the spin density. When comparing the macroscopic contributions to the IFE, related to the various photocurrents generated in the metal, the results are displayed in Figure 7d–i. Figure 7d, f, h represent, respectively, the total drift currents ***J**_**d**_*, magnetization currents ***J**_**M**_*, and ponderomotive currents ***J**_**Γ**_* in the nanoring, calculated using equations (6)–(8). Figure 7e, g, i depict the Z-component of magnetic field distributions at the surface of the structure associated with the currents in Figure 7d, f, h, respectively. As expected, for the drift currents, two counter-propagating photocurrents are present in the metal edge, which give rise to the magnetic field distribution, featuring a skyrmionic topology. Interestingly, these drift currents are localized exclusively at the inner and outer edges of the structure, with negligible contributions within the metal itself. Similarly, the magnetization currents ***J**_**M**_* exhibit the same symmetry as the drift currents ***J**_**d**_* at the structure’s edges. However, the resulting magnetic field distribution does not produce a skyrmionic topology. Instead, the magnetic field transitions from positive at the center to negative toward the outer edge and back to positive again. This behavior arises from the significant contribution of magnetization currents within the metal, which influence the overall field distribution differently.

In contrast, as seen in the case of a thin metal layer excited by a Gaussian beam (see Supplementary information), the ponderomotive currents exhibit a symmetry opposite to that of the magnetization currents. This reversal leads to a distinctly different magnetic field distribution, one that supports a skyrmionic topology. In this scenario, the magnetic field is positive at the center and transitions to negative as it approaches the outer regions.

The calculation of the total magnetic moments generated by these contributions provides further insight. Specifically, the magnetic moment, define as $m=\frac{r\times J}{2}$ , due to the magnetization currents ***J**_**M**_* is, as expected, exactly equal to the magnetic moment **M** of the magnetization itself (−4.7767e^−37^ A m^2^). On the other hand, the magnetic moment due to the ponderomotive currents ***J**_**Γ**_* has an opposite sign and a greater amplitude than that of the magnetization (1.4562e^−36^ A m^2^), resulting in a total magnetic moment of 9.8895e^−37^ A m^2^, equal to the one calculated from the drift currents ***J**_**d**_*.

These results are significant for several reasons. First, depending on the structure studied – whether a metal layer (see Supplementary information), a disk (see Supplementary information), or a gold nanoring – the amplitudes and signs of the magnetizations, currents, and magnetic moments change dramatically. Additionally, these results demonstrate the versatility in manipulating IFE-related effects at the nanoscale in plasmonic structures. Finally, while in the case of a uniform gold layer the primary contribution comes from the magnetization, in this scenario, it is the ponderomotive forces that dominate. Therefore, the best approach to account for all these contributions, regardless of the plasmonic nanostructure considered, is to include all drift photocurrents (***J**_**d**_*) when estimating the overall effect of light on magnetization and the generation of a magnetic field via the IFE.

## 3 Conclusions

In conclusion, our theoretical investigation has demonstrated that the manipulation of light, specifically its polarization in the near-field of a plasmonic nanostructure, allows for the generation of a vectorial magnetic field distribution exhibiting the topology of a Neel-type skyrmion with a skyrmion number of ±1. This novel observation arises from exploiting the dark (or anti-bonding) mode in a ring-shaped gold nanostructure, allowing the generation of two counter-propagating drift currents in the inner and outer segments of the ring. We established that employing this dark mode enables the generation of identical elliptical polarizations on each side of the metal, giving rise to the counter-propagating currents. Furthermore, we demonstrated that a diverse set of parameters can be employed to achieve this dark mode and, consequently, the vectorial distribution characteristic of a skyrmion.

Ultimately, this distribution was illustrated through a case study illustrating the reversal of the magnetic field orientation in the spatial vector distribution from the center to the end of the plasmonic structure. This behavior aligns well with the anticipated characteristics of this skyrmion symmetry. Finally, the breakdown of this effect into the various components of the drift currents highlights the intricate mechanisms involved. It also emphasizes that the most effective strategy for controlling the magnetization of a nanostructured plasmonic metal is to account for the total drift currents, providing a more comprehensive understanding of the phenomenon.

The findings presented in this study represent a significant advancement in the generation and manipulation of nanoscale magnetic field distributions by IFE. This breakthrough opens avenues for directly implementing skyrmionic topological structures in magnetic materials through an all-optical approach, potentially operating at ultrafast timescales. Consequently, this work holds promise for applications in various fields, including manipulating magnetic processes, ultrafast magnetic modulation, magnetic trapping, spin currents, and spin precession, with direct applications such as ultrafast data writing and processing.

## Supporting information

Comparison of optical properties of isolated nanostructures providing the nanoring. Comprehensive information on the divergent polarization between the two bonding and anti-bonding modes. Dipolar study of bonding and anti-bonding modes. Distribution of electric fields in the nanoring for bonding and anti-bonding modes at different times in an optical cycle.

## Supplementary Material

Supplementary Material Details
