# Supplementary material for: From dark modes to topology: light-induced skyrmion generation in a plasmonic nanostructure through the inverse faraday effect
Source: Nanophotonics. 2025 Jun 18;14(14):2453–62. doi: 10.1515/nanoph-2025-0096 (PMC12273543; doi:10.1515/nanoph-2025-0096)
Supplement: Supplementary file 1 — Supplementary Material Details [file j_nanoph-2025-0096_suppl_001.pdf]

Supporting Information

From Dark Modes to Topology: Light-Induced Skyrmion Generation in a Plasmonic Nanostructure Through the Inverse Faraday Effect

Xingyu Yang[1], Ye Mou[2], Bruno Gallas[1], Sébastien Bidault[3], and Mathieu Mivelle[1, *]

[1]Sorbonne Université, CNRS, Institut des NanoSciences de Paris, INSP, F-75005 Paris, France

[2]School of Electronic and Information Engineering, Ningbo University of Technology, No. 201, Fenghua Road, Jiangbei District, Ningbo, Zhejiang, China

[3]Institut Langevin, ESPCI Paris, Université PSL, CNRS, F-75005 Paris, France

*Corresponding authors:

mathieu.mivelle@sorbonne-universite.fr

Keywords: skyrmion, plasmonic nanoantenna, inverse Faraday effect, nanophotonics, light matter interactions

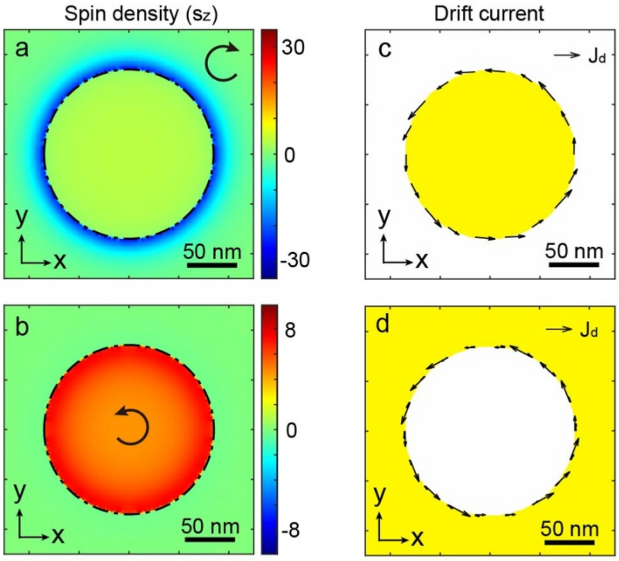

Figure S1. Comparison of optical properties of isolated nanostructures providing the nanoring: nanopatch and nanohole for a 30 nm gold thickness, deposited on a glass substrate, and for an excitation at λ = 754 nm and 825 nm with right circular polarization, respectively. Spatial distribution of spin densities in an XY plane at the center Z of a) a nanopatch and b) a nanohole. The arrows represent the helicity of light near the antennas. An important observation here is that, for the same excitation polarization of these plasmonic nanostructures, the local light polarization is opposite. c) and d) Spatial distribution of drift currents associated with the spin densities shown in a) and b), respectively. The length of the arrows represents the relative amplitude of drift currents.

<u>Comprehensive information on the divergent polarization between the two bonding and anti-bonding modes.</u>

As illustrated in Figures 2 and 4, the direction of the drift currents is associated with the z component of spin density. The spin density itself characterizes the handedness of the local elliptically polarized light, and the local polarization state, or the nearfield electric field, is directly influenced by the charge distribution in Figure 3c,d. Notably, the two intrinsic modes exhibit entirely different charge distributions.

In the case of the bonding mode, depicted in Figure S2a, it is composed of two parallel electric dipoles (ED). Each ED exhibits a spider-like electric field distribution in the near field. Consequently, the total electric field results from the contribution of both EDs, producing the same electric field distribution as shown in Figure S2a. To assess the handedness of the

nearfield, the orientation of the electric field at different times is indicated by black arrows in Figure S2a(ii, iv). Considering that the nano-ring is excited by right-handed circularly polarized (RHCP) light, the charge distribution and electric field undergo a 90-degree rotation after a quarter time period, as seen in Figure S2a(i,ii) and Figure S2a(iii,iv). In a complex representation, the electric field distribution in Figure S2a(i,ii) and Figure S2a(iii,iv) can be treated as the real and imaginary parts of the electric field. The change of orientation between Re(E) and Im(E) (alternatively between t_0 and t_0+T/4) reveals the handedness of local elliptical light, as depicted in Figure S2b. This distribution is further validated by numerical simulation results from Lumerical FDTD. Figure S3(a,b) and Figure S3(c,d) display the real (Re(E)) and imaginary (Im(E)) parts of the distribution at 1100 nm (bonding mode), respectively. The opposite handedness inside and outside the nano-ring aligns with the spin density distribution in Figure 4b.

A similar analysis is applied to the anti-bonding mode, but it is more intricate due to opposing EDs in Fig S2c. In this case, the total electric field combines contributions from the inner (E_{inner} - red) and the outer (E_{outer} - blue) ED. These two EDs exhibit destructive interference in the near field, and the orientation of the total electric field is determined by the relative strength of local E_{inner} and E_{outer}. The strength of local E_{inner} and E_{outer} depends on the intensity of the ED and the distance to each ED source. According to the spectrum of charge density in Figure 3b, the anti-bonding mode appearing at 570 nm has a relatively stronger inner ED and a weaker outer ED. Consequently, E_{inner} is stronger than E_{outer} at positions farther from the two ED sources, which are the top, bottom, and middle positions in Figure 2c(i). Here, the solid line indicates a stronger local electric field, while the dashed line indicates a weaker local electric field. As for the left and right positions in Figure S2c(i), they are situated next to the outer ED. In this small region, E_{outer} will be stronger than E_{inner}, benefiting from the shorter distance to the source. As a result, the total electric field has a nearly homogeneous distribution in the near field, as shown in Figure S2c(ii). Subsequently, the total electric field undergoes a 90-degree rotation after a quarter time period considering RHCP incident light, as depicted in Figure S2c(iii and iv). Figure S3(e,f) and Figure S3(g,h) display the real (Re(E)) and imaginary (Im(E)) parts of the distribution at 570 nm (anti-bonding mode), respectively. The homogeneous handedness inside and outside the nano-ring aligns with the spin density distribution in Figure 4a

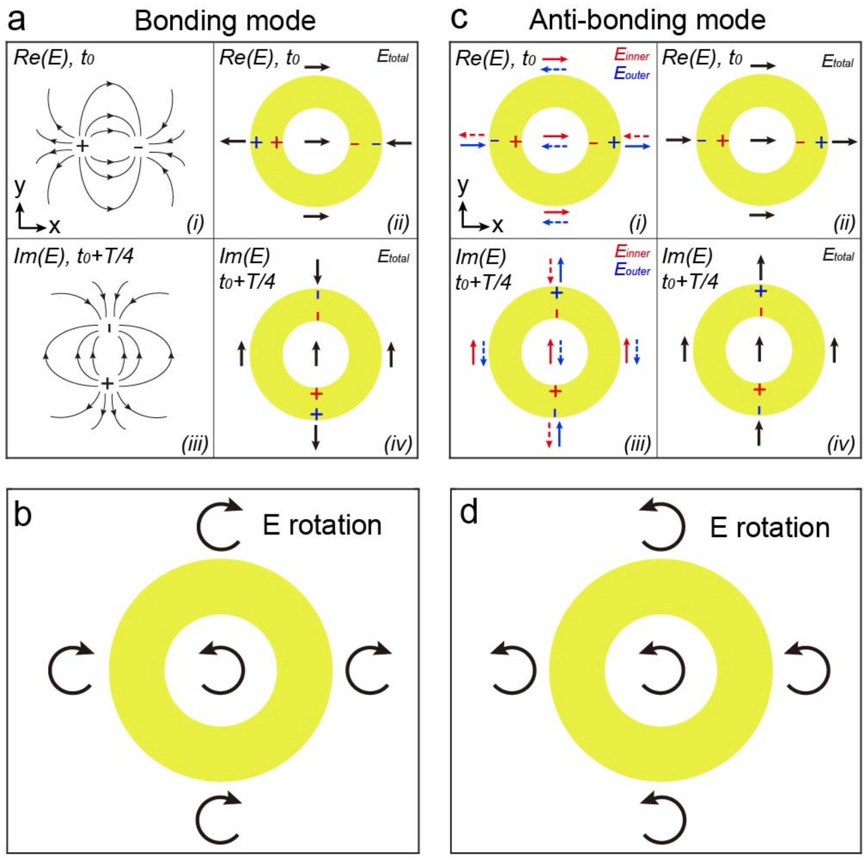

Figure S2. Dipolar study of bonding and anti-bonding modes. a) Spatial orientation in an XY plane of the electric field for i) a dipole oriented along X, ii) the nanoring excited by right circular polarization at t_0 and at the wavelength of the bonding mode, iii) an electric dipole oriented along Y, and iv) the nanoring excited by right circular polarization at $t_0 + T/4$ and at the wavelength of the bonding mode. b) Local polarization resulting from excitation of the nanoring by right circular polarization for the bonding mode. The arrows represent the helicity of light. c) Spatial orientation of the electric field resulting from the coupling between two opposite dipolar modes for excitation by right circular polarization of the nanoring at the wavelength of the anti-bonding mode at i, ii) t_0 and iii, iv) $t_0 + T/4$. In i) and iii), the red arrows represent the contribution of the inner dipole of the nanoring, the blue arrows represent that of the outer dipole, and the solid arrows represent the main contributions. In ii) and iv), the black arrows represent the orientation of the total electric field once the contributions of each dipole are taken into account. d) Local polarization resulting from excitation of the nanoring by right circular polarization for the anti-bonding mode. The arrows represent the helicity of light.

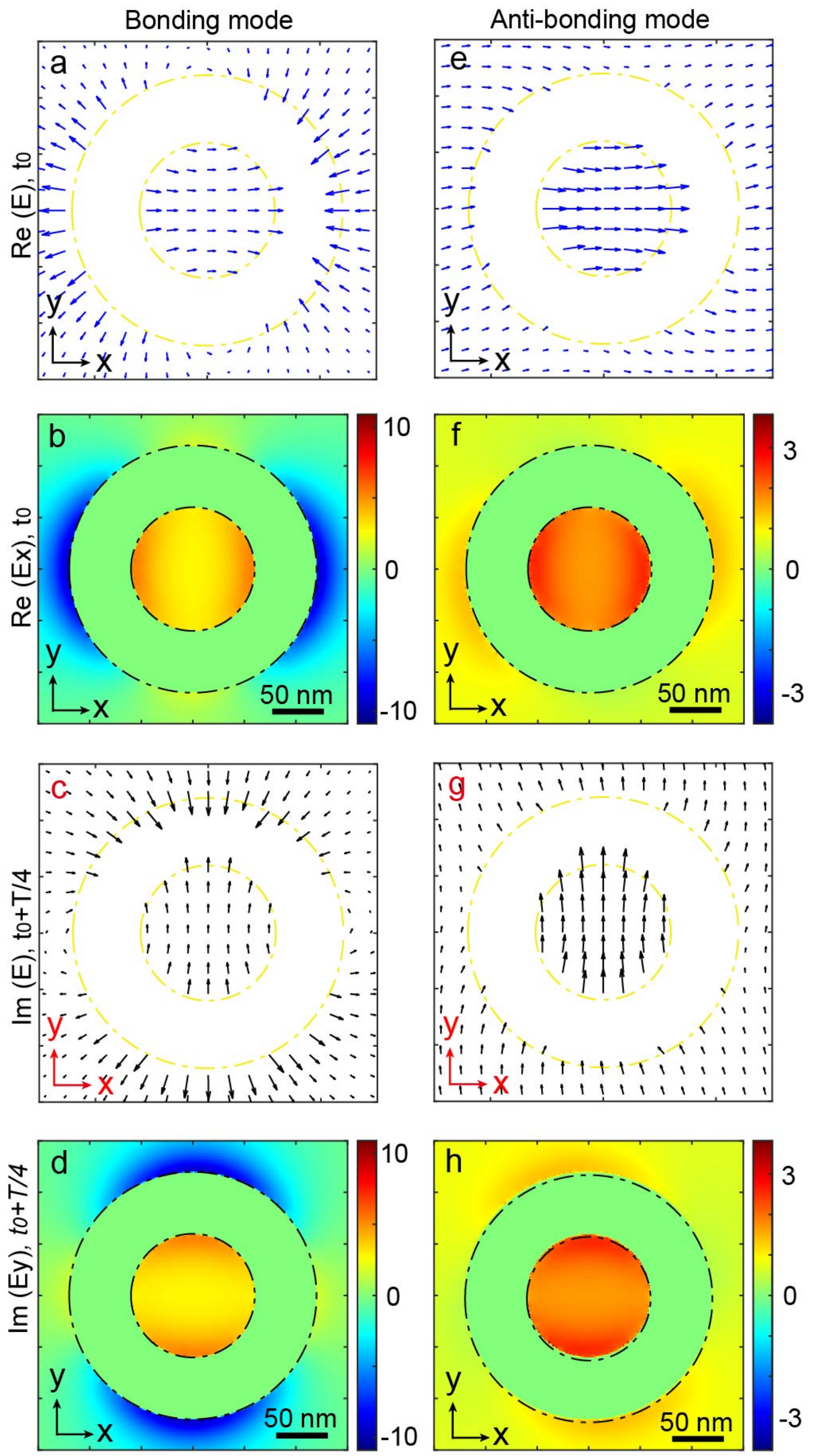

Figure S3. Distribution of electric fields in the nanoring for bonding (λ = 1100 nm) and anti-bonding (λ = 570 nm) modes at different times in an optical cycle and excited by right circularly polarized light. a, c) Vectorial distribution and b, d) electric field components in an XY plane at the center Z of the nanoring at a, b) $t=t_0$ and c, d) $t=t_0 + T/4$ for the bonding mode. e, g) Vectorial distribution and f, h) electric field components in an XY plane at the center Z of the antenna for times e, f) $t = t_0$ and g, h) $t=t_0 + T/4$ in the case of the anti-bonding mode. The length of the arrows represents the relative amplitude of electric field.

Study of the Different Contributions to the IFE:

We have conducted an in-depth analysis to rewrite and re-explain the different optical contributions to the IFE in plasmonic nanostructures. It should be noted that no smoothing algorithms were applied to any of the results presented in the manuscript or in the Supplementary Information.

Let us begin by addressing the equations that govern the generation of magnetization in a metal through optical excitation.

To address this, we will begin by utilizing the continuity equation. The continuity equation describes the transport or conservation of a physical quantity. In classical electrodynamics, it specifically governs the conservation of free electrons, which is expressed as:

$$\frac{\partial n}{\partial t} + \nabla \cdot (n\mathbf{v}) = 0 \qquad (1)$$

By introducing the elementary charge (*e*) on both sides, we obtain:

$$e\frac{\partial n}{\partial t} + \nabla \cdot \mathbf{J_{cd}} = 0 \qquad (2)$$

As a result, there are fluctuations in the free electron density over time, caused by the flow of conduction current ($\mathbf{J_{cd}} = en\mathbf{v}$) in space. Here, we decompose the electron density into a time-averaged component, $\langle n \rangle$, and a fluctuating component, (δn)

$$n = \langle n \rangle + \delta n \quad (3)$$

Since in the electron density (*n*), only the fluctuating component (δn) is time-dependent,

$$e\frac{\partial(\delta n)}{\partial t} + \nabla \cdot \mathbf{J_{cd}} = 0 \quad (4)$$

Similarly, in the case of a time-harmonic field, Eq. (2) becomes:

$$e(-j\omega)\delta n + \nabla \cdot \mathbf{J_{cd}} = 0 \qquad (5)$$

As a result, we obtain:

$$\delta n = \frac{1}{j\omega e}\nabla \cdot \mathbf{J_{cd}} \qquad (6)$$

It is this fluctuating part of the charge density (δn) that plays a crucial role in the theory of the IFE. Hence

$$\mathbf{J} = e(\langle n\rangle + \delta n)\mathbf{v} \qquad (7)$$

The first part describes the conduction currents (J_{cd}), which dominate in (**J**). Therefore, the electron velocity can be approximated as:

$$\mathbf{v} = \frac{\sigma}{e\langle n\rangle}\mathbf{E} \qquad (8)$$

By substituting (δn) in Eq. (7) with the expression from the continuity equation in Eq. (6), we obtain the second part of (**J**):

$$e(\delta n)\mathbf{v} = e(\frac{1}{j\omega e}\nabla\cdot\mathbf{J_{cd}})\mathbf{v} = e(\frac{1}{j\omega e}\nabla\cdot\mathbf{J_{cd}})(\frac{\sigma}{e\langle n\rangle}\mathbf{E}) \qquad (9)$$

It is important to note that both $\mathbf{J_{cd}}$ and $\mathbf{E}$ are expressed in complex form, where only the real part of each quantity has physical significance. Both are time-dependent, with a common factor exp(−jωt), resulting in a time-independent component:

$$\begin{aligned} e\langle\delta n\cdot\mathbf{v}\rangle &= \frac{e}{4}(\delta n\cdot\mathbf{v}^* + \delta n^*\cdot\mathbf{v}) \\ &= -\frac{j}{4e\langle n\rangle\omega}[\mathbf{J_{cd}}^*(\nabla\cdot\mathbf{J_{cd}}) - c.c.] \qquad (10) \\ &\textit{where} \quad \mathbf{J_{cd}} = \sigma\mathbf{E} \end{aligned}$$

This time-independent DC current is what we refer to as the drift current, denoted as $\mathbf{J_d} = e\langle\delta n\mathbf{v}\rangle$. Through a mathematical transformation for arbitrary vectors (**A**) and (**B**),

$$\nabla\times(\mathbf{A}\times\mathbf{B}) = (\mathbf{B}\cdot\nabla)\mathbf{A} - \mathbf{B}(\nabla\cdot\mathbf{A}) + \mathbf{A}(\nabla\cdot\mathbf{B}) - (\mathbf{A}\cdot\nabla)\mathbf{B} \qquad (11)$$

The drift current in Eq. (10) can be separated into two components.

$$\mathbf{J_d} = -\frac{j}{4e\langle n\rangle\omega}\nabla\times(\sigma^*\mathbf{E}^*\times\sigma\mathbf{E}) + \frac{1}{4e\langle n\rangle\omega}[j(\sigma^*\mathbf{E}^*\cdot\nabla)\sigma\mathbf{E} + c.c.] \quad (12)$$

These two contributions represent the magnetization currents and the ponderomotive currents, respectively. Thus, the drift current can be simply expressed as:

$$\mathbf{J_d} = \nabla\times\mathbf{M} + \mathbf{\Gamma} \quad (13)$$

Where:

- **M** is the magnetization,
- ∇×**M** represents the magnetization currents, and
- **Γ** represents the ponderomotive currents.

We now have a general equation that accounts for all the contributions to the inverse Faraday effect in metallic nanostructures. In this expression, ∇×**M** and **Γ** represent the macroscopic contributions responsible for the collective and steady motion of the metal's electrons (a direct current (DC)), while **M** represents the microscopic contribution linked to the circular motion of electrons around their center of mass.

Below, we propose to study these different contributions in two canonical cases: a thin gold film excited by a Gaussian beam (based on the study of R. Hertel), and the case of a gold nanodisk.

Case of the Gold Film:

This scenario allows us to identify the contributions mentioned by R. Hertel in his article *"Macroscopic drift current in the inverse Faraday effect"*. Here, a gold film of 30 nm thickness is excited at normal incidence by a Gaussian beam with a waist of 700 nm, right-hand circularly polarized, and at a wavelength of 700 nm (Figure S4a). From the spatial distribution of the electric field (Figure S4b), the magnetization **M**, the magnetization currents $\mathbf{J_M}$, and the ponderomotive currents $\mathbf{J_\Gamma}$ are calculated and shown in Figures R1c-e. As can be seen, the currents $\mathbf{J_M}$ and $\mathbf{J_\Gamma}$ are in opposite directions. Furthermore, if we

calculate the total drift currents $\mathbf{J}_d$ (Figure S4f) and compare them with the sum of the $\mathbf{J}_M$ and $\mathbf{J}_\Gamma$ currents, we observe the same current distribution, with a rotation direction similar to that of $\mathbf{J}_M$. This suggests that the value of the magnetization currents $\mathbf{J}_M$ is greater than that of the ponderomotive currents $\mathbf{J}_\Gamma$. To quantify these contributions, the total magnetic moment from each is calculated in each case.

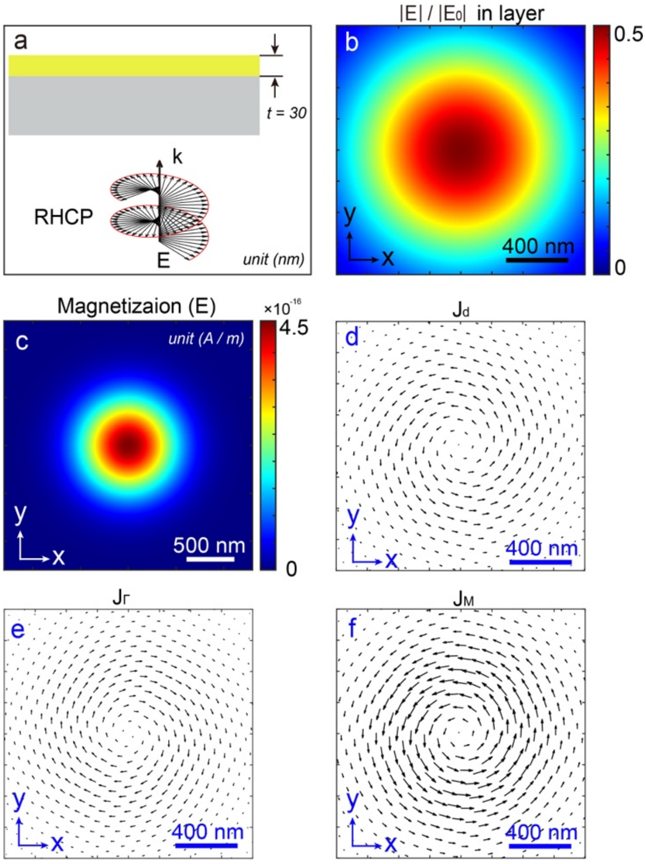

Figure S4. Thin gold layer (30 nm thick) excited by a Gaussian beam with a 700 nm waist, right-circularly polarized at a wavelength of 700 nm, under normal incidence. a) Schematic representation of the system. b) Distribution of the electric field amplitude in the central plane (Z) of the gold layer. Distribution of c) Magnetization **M**, d) magnetization currents $\mathbf{J}_M$, e) ponderomotive currents $\mathbf{J}_\Gamma$, f) total drift current $\mathbf{J}_d$ in the central plane Z of the gold layer.

The total magnetic moment due to magnetization **M** is equal to the spatial sum of the moments shown in Figure S4c and has a value of $1.474e^{-38}$A.m^2.

The total magnetic moment due to the magnetization currents $\mathbf{J}_M$, represented in Figure S4d, is calculated using the equation $\mathbf{m}_{J_M} = \frac{\mathbf{r}\times\mathbf{J}_M}{2}$ and has a value of $1.474e^{-38}$A.m^2.

The total magnetic moment due to the ponderomotive currents $\mathbf{J}_\Gamma$, represented in Figure S4e, is calculated using the equation $\mathbf{m}_{J_\Gamma} = \frac{\mathbf{r}\times\mathbf{J}_\Gamma}{2}$ and has a value of -7.272e^{-39} A.m^2.

Finally, the total magnetic moment due to the drift currents $\mathbf{J}_\mathbf{d}$, represented in Figure 2c, is calculated using the equation $\mathbf{m}_{J_\mathbf{d}} = \frac{\mathbf{r}\times\mathbf{J}_\mathbf{d}}{2}$ and has a value of 7.468e^{-39} A.m^2.

From these values, several observations can be made. First, the magnetic moments due to magnetization **M** and the magnetization currents $\mathbf{J}_\mathbf{M}$ are identical. Second, the magnetic moments resulting from the magnetization **M** and the ponderomotive currents $\mathbf{J}_\Gamma$ are of opposite signs. Lastly, the magnetic moment from the ponderomotive currents is half the magnitude of that due to magnetization, in absolute values.

These observations allow us to draw a parallel with the analytical results from the work of R. Hertel's. In his article, R. Hertel separated the inverse Faraday effect into two contributions: one microscopic and one macroscopic. These two contributions had opposite signs, with the macroscopic contribution having half the amplitude of the microscopic one.

Thus, we can identify the primary source of magnetization as the microscopic contribution (**M**), while the secondary, counteracting source is related to the ponderomotive currents $\mathbf{J}_\Gamma$. Since the magnetization values from the microscopic contribution **M** and its associated currents $\mathbf{J}_\mathbf{M}$ are exactly equal, we can conclude that these two magnetizations represent two sides of the same coin, essentially forming the same contribution to the IFE in the metal. Finally, from this comparison, we can deduce that considering the total drift currents $\mathbf{J}_\mathbf{d}$ provides the overall response, encompassing both the micro and macroscopic contributions to the IFE in the metal.

Case of a Gold Nanodisk:

For this study, a gold nanodisk with a diameter of 150 nm and a thickness of 30 nm, placed on a glass substrate, was excited by a right-handed circularly polarized plane wave at a wavelength of 700 nm, incident from the negative Z direction (Figure S5a). As with the gold film, the various contributions to magnetism were calculated. Figures S5b-e respectively show the magnetization **M**, the magnetization currents $\mathbf{J}_\mathbf{M}$, the ponderomotive currents $\mathbf{J}_\Gamma$, and the total drift currents $\mathbf{J}_\mathbf{d}$.

As observed here, similar to the case of the gold thin film, the ponderomotive currents **J_Γ** at the disk's edge are oriented opposite to the magnetization currents **J_M**. To quantify the impact of each contribution, the corresponding magnetic moments have also been calculated.

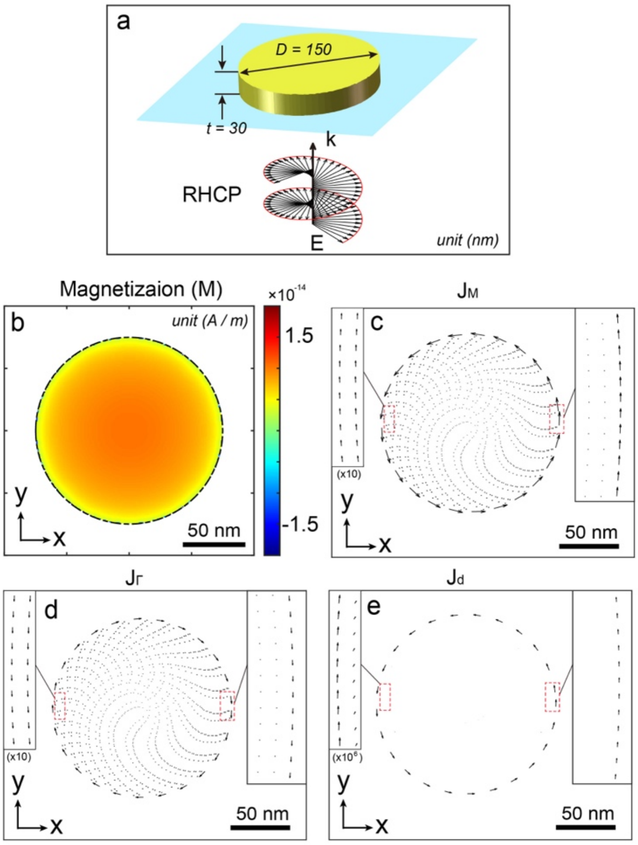

Figure S5. A gold nanodisk with a diameter of 150 nm and a thickness of 30 nm, placed on a glass substrate excited by a right-handed circularly polarized plane wave at a wavelength of 700 nm, under normal incidence. a) Schematic representation of the system. Distribution of b) Magnetization **M**, c) magnetization currents **J_M**, d) ponderomotive currents **J_Γ**, e) total drift current **J_d** in the central plane Z of the gold nanodisk.

The total magnetic moment due to magnetization is equal to the spatial sum of the moments shown in Figure S5b and has a value of 4.9113e^{-36} A.m^2.

The total magnetic moment due to the magnetization currents **J_M** , represented in Figure S5c, is calculated using the equation $\mathbf{m}_{J_M} = \frac{\mathbf{r}\times\mathbf{J}_M}{2}$ and has a value of 4.9113e^{-36} A.m^2.

The total magnetic moment due to the ponderomotive currents $\mathbf{J}_\Gamma$, represented in Figure S5d, is calculated using the equation $\mathbf{m}_{J_\Gamma} = \frac{\mathbf{r}\times\mathbf{J}_\Gamma}{2}$ and has a value of 7.1796e^{-36} A.m^2.

Finally, the total magnetic moment due to the drift currents $\mathbf{J}_\mathbf{d}$ (Figure S5e) is calculated using the equation $\mathbf{m}_{J_\mathbf{d}} = \frac{\mathbf{r}\times\mathbf{J}_\mathbf{d}}{2}$ and has a value of 1.1993e^{-35} A.m^2.

Once again, we observe that the magnetic moment arising from microscopic magnetization **M** and the corresponding magnetization currents $\mathbf{J}_\mathbf{M}$ are identical. However, as highlighted in this analysis, the distinct contributions of the Inverse Faraday Effect (IFE) to the overall magnetic moment differ significantly from those in the gold thin film case. In the thin film, the magnetic moments due to magnetization and ponderomotive contributions held opposite signs, whereas here, they share the same sign. This difference arises from the fact that the macroscopic currents within the metal and at the edges of the structure have opposite directions, both for the magnetization currents and the ponderomotive currents. These differences in orientation are due to the structural discontinuity at the metal edges. Inside the metal, the continuity of the fields and gradients determines the direction of the currents, whereas at the edges, the discontinuity in these quantities reverses their orientation. This discontinuity requires the use of an integral method to accurately evaluate this behavior (Jackson, John David, and Ronald F. Fox. "Classical electrodynamics." (1999): 841-842).

In this case, the contribution of the ponderomotive currents inside the metal to the magnetic moment is greater than that of the edge currents, resulting in a positive magnetic moment, similar to that produced by the magnetization currents. This leads to a cumulative contribution from these different effects, in contrast to the case of the gold layer. In contrast, for $\mathbf{J}_\mathbf{d}$ in Figure S5e, the currents within the structure are negligible, being six orders of magnitude smaller. As a result, the magnetic moment associated with $\mathbf{J}_\mathbf{d}$ can be determined with ease.

These findings are significant for several reasons. Firstly, as observed, the behavior of currents, magnetizations, and the resulting magnetic moment varies dramatically between systems. This suggests that the structural design should be chosen carefully based on the intended effect—whether generating a magnetic field or inducing a magnetic moment. Ultimately, the most effective approach to comprehensively understanding the behavior of

a plasmonic nanostructure is to consider the drift currents $\mathbf{J_d}$ in their entirety, without separating different components.

Additional plasmonic geometry capable of generating a skyrmionic topology:

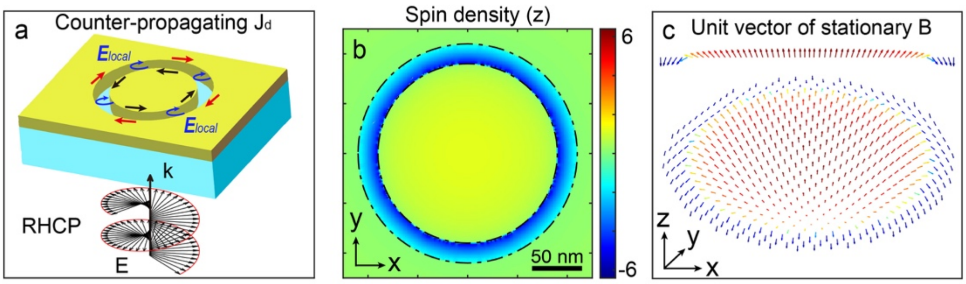

Figure S6. a) Coaxial nanoaperture with an inner diameter of 90 nm and an outer diameter of 110 nm milled into a 30 nm-thick gold film. The structure is illuminated by a right-handed circularly polarized plane wave at 660 nm. b) Inside the aperture, the optical field becomes uniformly left-handed elliptically polarized, driving counter-propagating drift currents in the inner and outer gold walls. c) These opposed currents produce, through the inverse Faraday effect, a magnetic field that exhibits a skyrmionic topology.
